# *In vitro* 3D Systems to Model Tumor Angiogenesis and Interactions With Stromal Cells

**DOI:** 10.3389/fcell.2020.594903

**Published:** 2020-11-05

**Authors:** Noémie Brassard-Jollive, Catherine Monnot, Laurent Muller, Stéphane Germain

**Affiliations:** ^1^Center for Interdisciplinary Research in Biology, College de France, CNRS UMR 7241, INSERM U1050, PSL Research University, Paris, France; ^2^Sorbonne Université, Collège Doctoral, Paris, France

**Keywords:** 3D micro-tumors, microfluidic, angiogenesis, tumor microenvironment, tumor–stromal cell interactions, personalized medicine

## Abstract

*In vitro* 3D culture systems provide promising tools for screening novel therapies and understanding drug resistance mechanisms in cancer because they are adapted for high throughput analysis. One of the main current challenges is to reproducibly culture patient samples containing cancer and stromal cells to faithfully recapitulate tumor microenvironment and move toward efficient personalized medicine. Tumors are composed of heterogeneous cell populations and characterized by chaotic vascularization in a remodeled microenvironment. Indeed, tumor angiogenesis occurs in a complex stroma containing immune cells and cancer-associated fibroblasts that secrete important amounts of cytokines, growth factors, extracellular vesicles, and extracellular matrix (ECM). This process leads to the formation of inflated, tortuous, and permeable capillaries that display deficient basement membrane (BM) and perivascular coverage. These abnormal capillaries affect responses to anti-cancer therapies such as anti-angiogenic, radio-, and immunotherapies. Current pre-clinical models are limited for investigating interactions between tumor cells and vascularization during tumor progression as well as mechanisms that lead to drug resistance. *In vitro* approaches developed for vascularization are either the result of engineered cell lining or based on physiological processes including vasculogenesis and sprouting angiogenesis. They allow investigation of paracrine and direct interactions between endothelial and tumor and/or stromal cells, as well as impact of biochemical and biophysical cues of the microenvironment, using either natural matrix components or functionalized synthetic hydrogels. In addition, microfluidic devices provide access to modeling the impact of shear stress and interstitial flow and growth factor gradients. In this review, we will describe the state of the art co-culture models of vascularized micro-tumors in order to study tumor progression and metastatic dissemination including intravasation and/or extravasation processes.

## Introduction

### Angiogenesis in the Tumor Microenvironment, a Multidimensional Process

Understanding the mechanisms that govern tumor initiation, development, and response to therapy is one of the most obvious and challenging tasks in our quest to fight cancer. Every cancer originates from a single cell but tumors rapidly progress as heterogeneous populations of cells, since single cells acquire genetic and phenotypic differences from each other during expansion of the neoplastic cell population. In addition, oncogenes and tumor suppressor genes tend to show strong context specificity that depends on the tumor type, the cell-of-origin, and the environmental factors (Schneider et al., 2017). The desmoplastic reaction of the stroma, consisting of basement membrane (BM), extracellular matrix (ECM), fibroblasts, immune cells, and vasculature, can distinguish some tumor entities from other tumor types, such as sarcomas, even if they are driven by the same oncogene (Tomlinson et al., 1999). These distinct cell types establish complex dialogs that comprise both homotypic and heterotypic interactions. In addition, these cells secrete growth factors and cytokines, synthesize, and remodel a complex ECM that exerts physical constraints. These biochemical and mechanical signals are major components of a specific tumor microenvironment that profoundly affects tumor progression by modulating tumor cell proliferation but also by inducing angiogenesis aimed at providing oxygen and nutrients. Indeed, solid tumors depend on angiogenesis for growth and metastasis and numerous efforts have therefore been undertaken in the last decades to develop tools that target angiogenesis, some of which have been clinically developed. Nevertheless, these drugs often lack associated biomarkers of response and efficacy and might also be associated with acquisition of drug resistance and therapeutic failure in a long-term perspective. Therefore, reconstituting such a complex process as cancer and tumor angiogenesis *in vitro* is still rather technically challenging and represents an unmet medical need that must be addressed.

### Current Angiogenesis Models and Their Limitations

Basement membrane extracts from Engelbreth-Holm-Swarm (EHS) mouse tumor cells, such as Matrigel^TM^, have been extensively used for the so-called “tube formation assay” in order to investigate angiogenesis (Arnaoutova et al., 2009). This easy-to-perform assay is the most widely used *in vitro* angiogenesis assay. It should, however, be understood that the tube-like or capillary-like *in vitro* assays using endothelial cells (EC) plated on top of BM extract are not considered as actual angiogenesis models by the community since neither their structure nor the mechanisms of their formation are physiologically relevant (Simons et al., 2015; Nowak-Sliwinska et al., 2018). Furthermore, many mesenchymal cell types, including fibroblasts and smooth muscle cells, also organize in networks in response to the matrix alignment generated by tension forces of cellular traction (Vernon et al., 1992). These assays are commercially successful but nevertheless insufficient to address the complexity of tumor angiogenesis and only made it more important to develop relevant 3D models. Whereas more advanced *in vivo* and *in vitro* models aimed at mimicking tumor angiogenesis have led to the discovery of novel therapies, most of these have nevertheless failed in clinical trials, thus shedding light on the numerous limitations of current pre-clinical models. Angiogenesis actually undergoes multiple discrete steps that can be individually evaluated and quantified by a large number of bioassays that have been reviewed elsewhere (Nowak-Sliwinska et al., 2018). In this review, we will focus on integrated assays aimed at properly reproducing the morphogenetic events of the formation of new capillaries.

## *In vitro* 3D Systems to Model Tumor and Stromal Cell Interactions With Capillaries

Diversification of *in vitro* 3D culture methods including culture supports, cells, imaging, and quantification has led to a great diversity of models. Here, we will survey the existing 3D models and highlight those that are urgently needed in order to fill the gap between 2D models and animal models of human disease, and that could help the research community to address the high attrition rates in drug development and to fulfill the transition toward personalized medicine.

Relevant *in vitro* models of capillary formation recapitulate many of the steps of angiogenesis, including EC migration and proliferation, lumen formation, branching, and anastomosis (Nakatsu et al., 2003; Nakatsu and Hughes, 2008). Indeed, angiogenesis- and vasculogenesis-based methods allow the formation of functional capillaries displaying adherens and tight junctions containing VE-cadherin/β-catenin complexes and Zonula occludens-1 (ZO-1), respectively, as well as accurate apical-basal polarity characterized by the abluminal deposition of BM components including laminin and collagen IV (Nowak-Sliwinska et al., 2018; Marchand et al., 2019).

### 3D Assays of Capillary Formation

#### Endothelial Cells

The use of EC cultures for engineering capillaries remains an experimental challenge. The most commonly used cells are human umbilical EC (HUVEC) (Nowak-Sliwinska et al., 2018). Other primary sources of human EC are aortic or microvascular EC derived from various tissues, most frequently from skin, defined as human dermal microvascular EC (HDMEC). Endothelial progenitor cells (EPC) harvested as endothelial colony-forming cells (ECFC) from cord blood can also be used, but those from adult peripheral blood exhibit limited proliferation potential (Ferratge et al., 2017). Recently, Palikuqi et al. (2020) reported “reset” vascular EC that transiently express ETS variant transcription factor 2 (ETV2) and that self-assemble into vascular networks and arborized cancerous human colon organoids. Quite remarkably, only cells from human origin are used in relevant 3D models; while isolation and culture of mouse EC have been developed for years now, their ability to form capillaries has proven to be more challenging (Nowak-Sliwinska et al., 2018). As example, mouse EC from the spleen stroma were used to explore the tumor-EC crosstalk in a 3D scaffold (Furlan et al., 2018). These cells were organized in elongated tubule-like structures but barely formed capillaries. Furthermore, neither human nor murine tumor EC have been reported to generate capillaries in 3D models, whether co-cultured with tumor cells or not.

#### Sprouting Angiogenesis- and Vasculogenesis-Based Assays

Two capillary morphogenesis models performed in 3D recapitulate sprouting angiogenesis and vasculogenesis processes.

**FIGURE 1:**
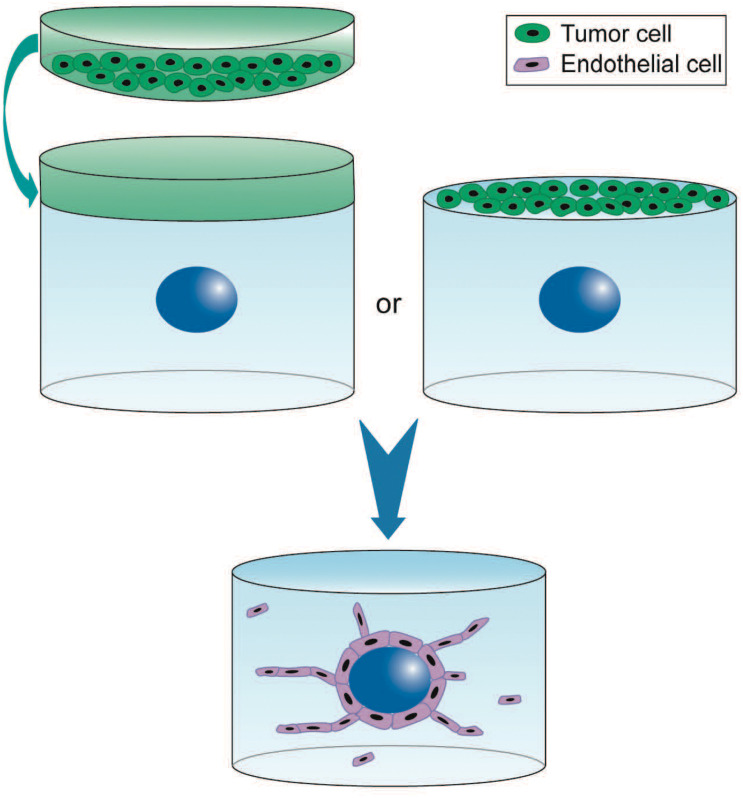
3D experimental settings for paracrine regulation of tumor cells on sprouting angiogenesis using the 3D angiogenesis assay. Endothelial cells are seeded on Cytodex beads (blue) and embedded in fibrin hydrogel. Culture is performed in presence of tumor cell conditioned medium (green) or tumor cells directly seeded on top of the hydrogel. EC proliferation and migration depend on the tumor paracrine regulation (Ramer et al., 2014). 3D cell culture is performed up to 6 days (blue arrow). This model is also used with endothelial spheroids. However, the absence of fibroblast contribution as much as the tumor paracrine effect is responsible for the chaotic structures often observed that barely form capillaries.

The bead and spheroid assays are relevant 3D models for investigating the mechanisms of sprouting angiogenesis (Nakatsu et al., 2003; Heiss et al., 2015). In the first assay, EC are seeded on polysaccharide beads before embedding in hydrogel and co-culture with fibroblasts to promote sprouting and formation of lumen-containing capillaries within a few days (Nowak-Sliwinska et al., 2018). These capillaries are led by tip cells, while their stalk cells are polarized and deposit BM. In the second assay, EC spheroids are embedded in a hydrogel that supports the formation of lumenized capillary-like structures where tip cells precede the elongation of individual sprouts originating from single spheroids (Heiss et al., 2015). These 3D models have been used for investigating the involvement of enzymes that remodel the microenvironment (Beckouche et al., 2015; Umana-Diaz et al., 2020) and factors secreted by tumor or stromal cells at several steps of sprouting angiogenesis (Welti et al., 2011; Ramer et al., 2014; Hernandez-Fernaud et al., 2017) (Figure 1). The impact of anti-angiogenic drugs is also increasingly explored using these models (Welti et al., 2011; Ramer et al., 2014; Heiss et al., 2015).

Vasculogenesis occurs via EC properties of self-assembling into a 3D vascular network. Single EC are seeded in collagen I hydrogels and cultured in presence of VEGF, FGF-2, or fibroblast-conditioned medium in order to allow formation of lumen-containing capillaries within 4–5 days (Nowak-Sliwinska et al., 2018). The 3D vasculogenesis assay is appropriate for studying interactions between tumor and EC and engineering vascularized micro-tumors (Chen et al., 2013, 2016; Kim et al., 2013; Bray et al., 2015; Jeon et al., 2015; Sobrino et al., 2016; Taubenberger et al., 2016). In addition, co-culture with stromal pericytes in the hydrogel recapitulates a key step of vascular morphogenesis consisting in their perivascular recruitment (Baudino, 2013). This assay is adapted to the study of tumor angiogenesis when isolated cancer cells or spheroids are co-embedded with sparse EC in the hydrogel. Interestingly, number and shape of capillaries can be tuned via modulating the density of the microenvironment, either in collagen or fibrin (see below, Ghajar et al., 2008).

### Biochemical and Biophysical Properties of Tumor Microenvironment Controlled in the 3D Gels

For engineering tumor microenvironment, a variety of natural and synthetic materials have been used as 3D scaffolds (Song et al., 2014). An important requirement for these scaffolds is their capacity to support simultaneously cancer cell proliferation/invasion and angiogenesis. Indeed, hydrogels have the ability to recapitulate many features of native ECM concerning their composition (either simple or composite), physical properties (stiffness, pore size, fiber length…), and biological functions (growth factors bioavailability, cell adhesion machinery…) (Li and Kumacheva, 2018). Modulation of these parameters does not only induce global changes of the 3D scaffold but also local modifications of the topology of cell microenvironment (Yamada and Sixt, 2019). Selection of the scaffold and associated culture medium is therefore a crucial step in engineering *in vitro* micro-tumors and their vasculature.

#### Natural Polymers for Hydrogels

Protein-based hydrogels consist in either purified single components of matrix proteins, including fibrin and collagen I or in crude extracts of natural matrices (Song et al., 2014; Li and Kumacheva, 2018).

Basement membrane extracts have been the most widely used biomaterial for many years, supporting growth for many tumor cell lines (Song et al., 2014). Assays combining 3D cultures of tumor spheroids overlayed with EC have thus also been performed (Benton et al., 2015) but do not faithfully reproduce angiogenesis (see above). Furthermore, even when used for actual 3D culture of EC, Matrigel^TM^ was demonstrated to induce formation of multicellular tubes that display an inversed luminal/abluminal polarization with deposition of BM components like laminin α4 in the lumen (Kuèera et al., 2009). Quite remarkably, using mixtures of Matrigel^TM^ with natural polymers like fibrin turned out to noticeably improve capillary formation and resulted in efficient inosculation with the host vasculature and further blood perfusion after implantation in mice (Laib et al., 2009). Recently, Palikuqi et al. (2020) developed matrices made of mixture of laminin, entactin, and collagen IV that support vasculogenesis.

Polymerization of fibrin occurs from fibrinogen cleavage by thrombin and is controlled by calcium ions, temperature, and fibrinogen concentration. Fibrin hydrogels have been widely used for investigating angiogenesis in 3D settings since their nano/macro fibrous architecture mimics native ECM. They provide robust support for both sprouting angiogenesis (Nakatsu et al., 2003; Ghajar et al., 2006, 2008) and vasculogenesis (Rao et al., 2012). Ghajar et al. (2006) reported that fibrin hydrogel, at the concentration of 2.5 mg/ml, induces capillary network formation. Using a bead assay, the same authors further demonstrated that increasing fibrin density from 2.5 to 10 mg/ml leads to a decrease in capillary network formation. Soft fibrin gels of 90 Pa-stiffness corresponding to low density at 1 mg/ml were optimal for cancer cell proliferation and spheroid formation when compared to stiffer gels (Liu et al., 2012). These studies thus indicate that the fibrin hydrogel provides a relevant 3D scaffold for studying *in vitro* micro-tumors and their vasculature.

Collagen I, the major protein of the interstitial ECM (Blache and Ehrbar, 2017), is a biocompatible and biodegradable material that possesses the intrinsic capacity to self-assemble into hydrogels under physiological conditions (Sorushanova et al., 2019). Collagen gels can also be produced using multiple cross-linking methods resulting in fibrous architectures similar to those of native ECM. *In vitro* studies have demonstrated that collagen gel density and porosity are modulated by pH, temperature, and collagen concentration through modulation of fibrillogenesis, therefore offering ways to control structural properties (Doyle et al., 2015; Sapudom et al., 2015). Collagen hydrogels provide bioactive microenvironments supporting cellular processes, such as cell adhesion sites, proteolytic-degradable sites, and ECM crosslinking (Laib et al., 2009). Several *in vitro* studies have demonstrated that collagen hydrogels support capillary network formation using 3D vasculogenesis or EC spheroid assays (Heiss et al., 2015; Blache and Ehrbar, 2017). Collagen I also supports invasion and growth of numerous tumor cell lines (Jeong et al., 2016). MDA-MB 231 breast cancer cells cultured within collagen hydrogel formed spheroids displaying necrotic and hypoxic core (Szot et al., 2011; Song et al., 2014). Using collagen I of various origin (bovine dermal and rat tail), Wolf et al. (2013) demonstrated that matrix metalloproteinase (MMP)-independent migration of tumor cells into 3D hydrogel depends both on deformation of the nucleus and scaffold porosity in response to space constraints without any correlation with scaffold stiffness.

Natural materials have numerous limitations, though. They suffer from variability, either batch-to-batch or according to species or tissue differences as well as purification protocols. They provide limited control of ECM compound concentration and presence of degradation- or adhesion-sites. They thus fail to provide full control of both matrix density and stiffness (Song et al., 2014).

#### Synthetic Hydrogel Materials

Among the many synthetic biomaterials used for 3D cell culture (Blache and Ehrbar, 2017), polyethylene glycol-heparin (PEG) is one of the most commonly used for the development of micro-tumors (Bray et al., 2015; Chwalek et al., 2015; Roudsari et al., 2016; Taubenberger et al., 2016). PEG hydrogel aims to mimic the natural ECM and to offer control of its design and synthesis (Lutolf and Hubbell, 2005; Blache and Ehrbar, 2017). Scaffolds are generated using multi-armed PEG and heparin crosslinked via cytocompatible Michael-type addition that enables cell embedding in 3D (Chwalek et al., 2015). A major advantage of these synthetic scaffolds is the possibility to decouple mechanical and biochemical properties. Indeed, increasing PEG (or PEG-heparin) concentration allows to control mechanical properties over a broad range, while maintaining the heparin concentration constant. In addition, PEG can be functionalized for (i) reversible binding of multiple growth factors (VEGF, FGF-2, SDF-1), (ii) covalent conjugation of adhesion ligands (RGD motifs), or (iii) proteolytic sites (Kraehenbuehl et al., 2008; Bray et al., 2015; Chwalek et al., 2015; Blache et al., 2016; Taubenberger et al., 2016; Blache and Ehrbar, 2017). These PEG hydrogels support capillary network formation *in vitro*. Indeed, in several studies, co-culture of EC and mesenchymal cells leads to formation of capillaries displaying lumens, BM deposition (laminin and collagen IV), and perivascular localization of mesenchymal cells (Blache et al., 2016; Blache and Ehrbar, 2017). These vessels recapitulate many features of angiogenesis *in vitro* and are similar to those formed in natural hydrogels. Moreover, PEG hydrogels support 3D tumor spheroid formation and growth of a hepatocellular carcinoma (HCC) cell line (Liang et al., 2011) and of various others such as breast, prostate, and lung cancer cell lines (Bray et al., 2015; Roudsari et al., 2016; Taubenberger et al., 2016). This 3D scaffold is therefore readily available for the *in vitro* study of micro-tumors and their vasculature.

### Static 3D Co-culture to Recapitulate Tumor–Stromal Cell Interactions With Capillaries

To date, multiple *in vitro* static 3D models have been engineered to recapitulate heterotypic interactions between tumor cells and capillaries. These are based on a large diversity of strategies using different scaffolds as well as tumor–stromal cell sources and methods for capillary formation. As described above, natural and synthetic scaffolds or tissue constructs are essential to support these cell interactions. Tumor cells are mainly human cell lines from breast, pancreatic, colorectal, skin, lung, and liver cancers cultured as single cells or in spheroids. Moreover, these models require stromal cells [e.g., fibroblasts, mesenchymal stem cells (MSC)] or stromal conditioned medium since tumor cells alone do not support capillary formation. Basically, two types of approaches have been performed using vascularized spheroids in 3D hydrogels or vascularized tissue constructs.

#### Vascularized Spheroids in 3D Hydrogels

##### Cellular interactions

**FIGURE 2:**
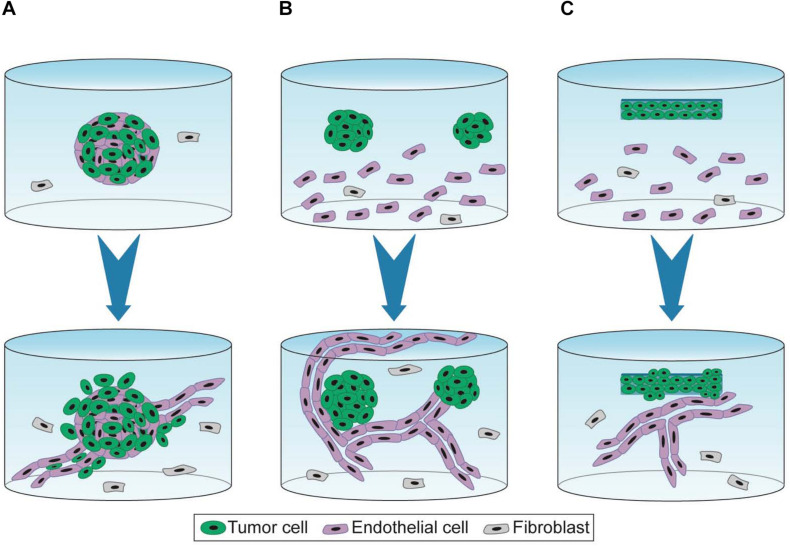
3D experimental settings of capillary formation and tumor growth. 3D cell co-culture is performed for 7 days **(A)**, 7–10 days **(B)**, and 10–21 days **(C)** (blue arrow). **(A)** Spheroids containing tumor and endothelial cells are embedded in fibrin hydrogel containing fibroblasts in suspensions. Capillaries are formed by a sprouting angiogenesis-mimicking process and grow away from the tumor mass (Ehsan et al., 2014). **(B)** Spheroid cells are embedded in functionalized PEG-derived hydrogel containing EC and fibroblasts in suspension. Capillaries formed by vasculogenesis, unfold the tumor mass but barely vascularize it (Bray et al., 2015; Taubenberger et al., 2016). **(C)** Dense artificial cancer mass (ACM) is obtained by plastic compression of collagen hydrogel containing tumor cells. ACM is then embedded in collagen hydrogel containing EC and fibroblasts in suspension. This setting is further compressed. Capillaries are formed by vasculogenesis (Magdeldin et al., 2017; Pape et al., 2019).

To recapitulate interactions between tumor cells and capillaries, Ehsan et al. developed vascularized tumor spheroids using various cancer cell lines from breast, lung, and colon. Multicellular spheroids containing tumor and EC were generated using the liquid overlay method and were co-embedded with isolated fibroblasts in fibrin hydrogels (Ehsan et al., 2014). The vascularized tumor spheroids exhibited sprouting angiogenesis, generating a capillary network that extends into the surrounding matrix within 7 days of culture (Figure 2A). In the case of colon cancer cell lines, capillaries localized within the spheroid have a distinct morphology and exhibit shorter, more branched, and irregular phenotype than capillaries that extend into the hydrogel. Interestingly, this model recapitulates specific pro-angiogenic capacities according to the type of tumor cell lines. Indeed, vascularized spheroids of breast tumor cell lines (MCF and MDA MB-231) exhibit more capillaries than those of lung or colon tumor cell lines. Quite surprisingly, co-seeding tumor cells, however, does not affect extent nor kinetics of sprouting and growth of the capillary network in the fibrin hydrogel. In addition, authors report that tumor cell-specific intravasation occurs in this model, as they detect SW620 cells localized and migrating in the capillary lumen, which is enhanced by low oxygen condition. It is, however, not determined whether presence of tumor cells in the lumen corresponds to actual trans-endothelial intravasation or engulfment of tumor cells upon initiation of capillary formation. This model nonetheless recapitulates tumor angiogenesis and interactions of tumor cells with capillaries in a fibroblast-containing hydrogel.

Bray et al. (2015) also investigated the interactions between tumor spheroids and capillaries by co-culture with MSC seeded in suspension in a synthetic hydrogel. First, spheroids were formed in hydrogel through proliferation of isolated tumor cells. After 7 days of culture, spheroids were harvested by collagenase treatment and seeded in a newly formed hydrogel containing both EC and MSC (Figure 2B). This protocol recapitulates the first avascular growing step of the tumor mass but requires a tough collagenase-based treatment for the co-culture model. Unlike the model described above (Ehsan et al., 2014), capillaries sprouted toward the tumor spheroids and did not invade the core of the spheroid but only established contacts at the spheroid surface (Bray et al., 2015).

Whereas both studies used spheroids devoid of invasive capacities, applying invasive spheroids in such models would be of major interest to monitor interactions between tumor protrusions and capillaries in the surrounding hydrogel in order to assess potential co-option processes.

##### ECM interactions

Models of vascularized spheroids in natural hydrogels effectively recapitulate cellular interactions within a 3D environment but are limited for studying interactions between tumor cells and specific ECM proteins. Cell–ECM interactions play a major role in tumor growth, invasion, and angiogenesis. ECM proteins such as collagen I, fibronectin, and laminin are major components of the stromal microenvironment (Provenzano et al., 2006; Schedin and Keely, 2011). As mentioned above, synthetic hydrogels functionalized with specific cell adhesion domains of these matrix proteins are also useful tools to recapitulate a wide range of ECM interactions. Taubenberger et al. (2016) investigated the involvement of specific cell adhesion domains on interactions between tumor cells organized in spheroids and capillaries. For this purpose, an integrin binding domain present in fibronectin and vitronectin (RGD), a laminin-111 derived adhesion peptide (IKVAV), and an integrin binding domain present in collagen I (GFOGER) were conjugated to PEG hydrogel. Tumor spheroid formation and co-inclusion with EC and MSC in these functionalized hydrogels were performed as in Bray et al. (2015). While interactions between tumor cells and capillaries were observed in each condition, both tumor spheroid invasiveness and tumor-EC interactions inside spheroids increased only in IKVAV and GFOGER functionalized hydrogels. This 3D culture model therefore constitutes an interesting tool to decipher the effect of ECM interactions on tumor cell growth, invasion, and angiogenesis.

Composition but also density of the ECM are crucial features that impact cell proliferation/invasion and angiogenesis. To modulate the ECM density, a strategy named plastic compression consists in removing water from collagen hydrogel containing tumor cells (Magdeldin et al., 2017; Pape et al., 2019). This dense artificial cancer mass (ACM) is then seeded in collagen hydrogel supplemented with laminin and containing fibroblasts and EC, thereby mimicking the stromal compartment, before further compression. ECM density modulated by plastic compression as well as ECM composition of the stroma modulated by laminin concentrations both regulate cancer cell invasion (Figure 2C). A high density of collagen I is associated with increased tumor cell invasion. Moreover, presence of laminin stimulated both tumor cell invasion and vascular network formation. Authors reported that EC seeded in the stromal compartment formed “healthy” and “tumorigenic” vascular networks in absence or in presence of colorectal tumor cells, respectively (Magdeldin et al., 2017). Pape et al. (2019) compared two colorectal tumor cell lines (HT29 and HCT116) displaying distinct invasive properties. Authors reported that HCT116 was more invasive than HT29, displaying an increased distance and surface area of invasion into the stromal compartment. Spheroids containing highly invasive cells form less complex and less branched vascular networks.

#### Vascularized Tissue Constructs

3D *in vitro* models described above recapitulate the crosstalk between tumor cells, stromal microenvironment, and vessels but none of them present a tissue-specific environment including both blood and lymphatic capillaries. Indeed, the vast majority of articles published up to now, aiming to recapitulate interactions between tumor cells and capillaries, focus on blood vessels. However, it is commonly accepted that lymphatic vessels are essential for tumor spread and metastasis (Stacker et al., 2014). Furthermore, formation of functional lymphatic capillaries has been documented for some years now either within 3D hydrogel (Montaño et al., 2010; Marino et al., 2014) or in skin constructs (Gibot et al., 2016, 2017) using HDMEC or lymphatic EC (LEC) isolated from human foreskin. These capillaries are positive for Prox1 (lymphatic-specific transcription factor) and CD31 staining thereby confirming their lymphatic nature. To recapitulate specific interactions between tumor cells and lymphatic capillaries, stromal tissues as well as skin constructs were engineered by the layer-by-layer technique (Bourland et al., 2018; Nishiguchi et al., 2018). Using this approach, the authors investigated: (i) intravasation into lymphatic capillaries, (ii) MMP secretion profiles, (iii) response to therapies.

The stromal tissue developed by Nishiguchi et al. is composed of fibroblasts synthetizing their own ECM and of EC/LEC self-assembled in blood and lymph capillaries as originally described by Decher (1997). Single cancer cells are seeded onto these tissues to form primary micro-tumors. For studying intravasation in blood or lymphatic capillaries, pancreatic and colorectal cell lines displaying hematogenous (MiaPaCa-2 cells) or lymphogenous metastasis (BxPC3 cells) were used. The specificity of these cell lines for each type of vessel was indeed reproduced *in vitro* (Nishiguchi et al., 2018). Interestingly, tumor cells displayed distinct MMP secretion profiles according to their invasive properties and target vasculatures: hematogenous cells secreted MMP-1, -2, -3, and -10 while lymphogenous cells secreted MMP-1, -2, and -9. This model is thus relevant for recapitulating specific interactions of tumor cells displaying hematogenous and lymphogenous metastasis properties, tumor invasion, and intravasation/extravasation.

To mimic the melanoma microenvironment, Bourland et al. (2018) engineered a skin construct using the self-assembly method. This model mimics the human skin morphology characterized by a dermis enriched in ECM components, a differentiated epidermis composed by a proliferative basal layer and multiple suprabasal layers resulting in a *stratum corneum*. Microvascular EC self-organized to form two distinct capillary networks, one of lymphatic vessels positive for podoplanin and another of blood vessels positive for CD31 but negative for podoplanin. Melanoma spheroids integrated at the dermo-epidermal junction invaded the epidermal layer and localized in close contact with lymphatic capillaries. Chronic treatment with Vemurafenib (Zelboraf^®^), a selective inhibitor of BRAF frequently used to treat melanoma bearing the BRAF-V600E mutation, affected tumor cell proliferation and apoptosis, thereby recapitulating changes in tumor morphology, without leading to any toxicity in the surrounding tissue. Interestingly, some tumor cells localized in close contact with fibroblasts continue to proliferate even after 12 days of treatment suggesting an effect of the microenvironment on therapeutic resistance.

All in all, these approaches better recapitulate the global tissue organization than vascularized spheroids in 3D hydrogel.

#### Conclusion

Culture of vascularized tumor spheroids in hydrogel is suitable to mimic interactions between tumor cells and capillaries. Moreover, using functionalized synthetic scaffolds, 3D hydrogel co-culture allows to study the impact of ECM proteins on tumor angiogenesis. Tissue constructs recapitulate the global tissue organization to create a more complex microenvironment than 3D culture in hydrogel. Figure 1 illustrates some 3D experimental settings of capillary formation and tumor growth. Table 1 is summing up the main features and applications of the static 3D models described above. Improvement of these models will consist in inclusion of other stromal cell types. Indeed, while fibroblasts are the major cell type used in these models, many other stromal cells such as immune cells, stem cells, or cancer associated fibroblasts (CAF) could be incorporated to recapitulate more complex features of the tumor microenvironment.

**TABLE 1:** *In vitro* 3D vascularized tumors in non-perfused models and applications.

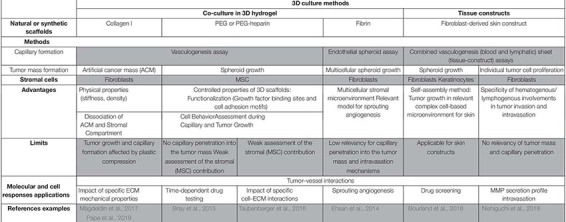

In addition, it is of major interest to include pericytes in these vascularized tumor spheroids to investigate the perivascular coverage defect observed in cancer. Other major factors involved in the vascularization process are shear forces and blood flow. These parameters require the use of microfluidic devices in order to be investigated *in vitro.*

### Multiple Microfluidic Angiogenesis Models

Over the last decade, microfluidic organs-on-chips have been extensively developed to model cancer (Zervantonakis et al., 2012; Sontheimer-Phelps et al., 2019). These tumor-on-a-chip recapitulate the multicellular architecture, tumor–tissue interface, and physical microenvironment of cancers growing within human organs. Furthermore, a unique feature of microfluidic systems is perfusion of the vascular compartment, allowing the study of shear forces and blood flow in tumor vascularization (Haase and Kamm, 2017). Advantages of these systems are high spatial and temporal control over cell patterning, chemical gradients, and mechanical stimuli. In these tumor-on-chip technologies, engineering perfused micro-vessels has been a major challenge addressed by several *in vitro* approaches, such as EC lining-, vasculogenesis-, or angiogenesis-based methods (Haase and Kamm, 2017; Wang et al., 2018; Sontheimer-Phelps et al., 2019). Microfluidic tumor-on-a-chip models have been further developed to study tumor cell interactions with the vasculature as well as intravasation/extravasation processes.

#### Engineering Perfused Micro-Vessels

##### EC lining method

The EC lining method is based on endothelial monolayers established on the inner walls of microchannels (Sudo et al., 2009; Shin et al., 2012; Zhang et al., 2012; Tourovskaia et al., 2014; Pauty et al., 2018; Andrique et al., 2019). The most widely described microfluidic platform comprises hydrogel-incorporating chambers between surface-accessible microchannels made of polydimethylsiloxane (PDMS) and coated with ECM proteins (Shin et al., 2012). In other devices, microchannels are made of hydrogel which require the inclusion of a cylindrical rod into collagen I and its removal after polymerization to form a microchannel for infusion of EC (Tourovskaia et al., 2014; Wong and Searson, 2014). EC lining method is applied to explore processes of intravasation and extravasation by tumor cells under combination of multiple controllable biochemical and biophysical microenvironment parameters (Zervantonakis et al., 2012; Bersini et al., 2014; Wong and Searson, 2014). These models provide some advantages over common static assays used for studying migration in response to chemotactic gradients, such as Boyden chamber and wound assay as they allow integration of multiple microenvironmental factors (cells, secreted factors, 3D ECM, flow).

##### Angiogenesis-based method

In microfluidic systems, angiogenesis is induced from either EC monolayers or pre-existing vessels (Haase and Kamm, 2017). A typical microfluidic platform, initially developed by Song and Munn (2011), contains a central chamber incorporating a hydrogel that is in direct contact with two surface-accessible microchannels. The EC infused in these microchannels cover the surface of the central hydrogel. This microfluidic device incorporates features such as: (i) contact between the abluminal side of EC and the hydrogel to allow angiogenic sprouting; (ii) fluid flowing through two adjacent channels and controllable fluid convection through the hydrogel; (iii) co-culture with other cell types seeded either in the central hydrogel or in the opposite channel to EC; (iv) specific growth factors gradients. This microfluidic platform has given rise to many proximate designs that are now commonly used by many research groups (Yeon et al., 2012; Abe et al., 2019; van Duinen et al., 2019).

##### Vasculogenesis-based method

As previously described, vasculogenesis-based method produces microcirculation by formation of interconnected lumens via a process that relies on the capacity of EC to self-assemble into capillaries under proper culture conditions and stimulation (Haase and Kamm, 2017; Wang et al., 2018). The model contains one central hydrogel seeded with EC in suspension and bordered by micro-fabricated channels for medium perfusion (Shin et al., 2012; Kim et al., 2013; Moya et al., 2013; Wang et al., 2017). Stromal cells such as fibroblasts (Chen et al., 2013, 2016; Sobrino et al., 2016), MSC, osteoblasts-differentiated cells (Jeon et al., 2015), or perivascular cells (Sobrino et al., 2016) are seeded either with EC in the central hydrogel region or in the surrounding channels.

This method allows to engineer capillaries that are not only properly polarized and lumenized but also functional. Indeed, perfusion of the vascular network with FITC reveals its barrier integrity and therefore allows investigation of regulatory mechanisms of vascular permeability (Sobrino et al., 2016). In this respect, only a few microfluidic models, however, include pericytes wrapping the abluminal surface of the endothelium even though pericyte coverage is required for establishment of vascular permeability *in vivo* (Kim et al., 2013; Sobrino et al., 2016). Thus, this represents an important challenge for the development of physiologically relevant models.

#### Strategies to Recapitulate Tumor–Vasculature Interactions

##### Culture in separate compartments

###### Interstitial and blood flow

There is a distinction between the functional perfusion of the entire microvascular networks (Kim et al., 2013; van Duinen et al., 2019) and interstitial flow applied in the microfluidic device without perfused capillaries (Abe et al., 2019). Microfluidic devices allow investigation of specific mechanical factors consisting in forces generated by fluid flow through either the hydrogel or the microvascular network, respectively, mimicking the interstitial (Abe et al., 2019) and the blood flow (Jeon et al., 2015). For example, in the single gel channel device of Song and Munn (2011) described above, convective flow through media channels results in interstitial flow across the hydrogel region. These authors demonstrated that interstitial flow enhanced sprouting morphogenesis consisting in increased formation of filopodia extending against the flow (Song and Munn, 2011). However, it remains unclear whether interstitial flow magnitude can be optimized to control 3D vascular network formation in combination with various VEGF concentrations. Therefore, using a wide range of interstitial flow in a microfluidic device dedicated to angiogenesis, Abe et al. (2019) demonstrated a pro-angiogenic effect of the interstitial flow that is not substituted by increasing VEGF concentration. Indeed, density and length of the microvascular network as well as lumen diameter of capillaries and area of degraded gel increased specifically in response to flow magnitude. This study is in agreement with previous work performed on avian embryos where interstitial flow has been reported to affect VEGF distribution in the mesenchyme (Ghaffari et al., 2017). In tumor, interstitial fluid pressure is high due to plasma leakage from blood capillaries and non-functional lymphatic networks which limit drug delivery (Viallard and Larrivée, 2017). Microfluidic approaches are thus powerful tools to study the effect of interstitial pressure within the tumor microenvironment, but no study has been performed using lymphatic vessels so far, to the best of our knowledge.

Blood flow through capillaries results in shear stress, which is the major biomechanical factor depending on fluid flow (Haase and Kamm, 2017; Wang et al., 2018). Shear flow can be applied using EC lining, angiogenesis-, and vasculogenesis-based methods. For the EC-lining based method, it is easy to assess the shear stress applied on the capillary network because geometries of the device are known. However, for angiogenesis- and vasculogenesis-based methods, generating a network homogeneously perfused remains quite challenging, as much as modeling flow in a complex network produced by cell self-assembly. Kim et al. (2013) have demonstrated EC alignment in the direction of applied shear flow. Moreover, Jeon et al. (2015) revealed that capillaries under laminar flow displayed decreased vasculature permeability and tumor cell extravasation rates when compared to static conditions.

###### Paracrine factors secreted by tumor cells

Some *in vitro* microfluidic platforms are engineered to study the effect of factors secreted by tumor and stromal cells on angiogenesis using growth factor cocktails (van Duinen et al., 2019) or co-culture of cells in separate channels (Kim et al., 2013; Miller et al., 2018). In contrast to static culture, these models integrate both perfusion and generation of robust biomolecular gradients (van Duinen et al., 2019). For example, van Duinen et al. (2019) perfused a microfluidic device by passive leveling using a rocker platform thus applying the flow through all 40 units and resulting in reproducible gradient formation. They identified a combination of pro-angiogenic factors VEGF-A, and Sphingosine-1-phosphate, together with the PKC activator Phorbol 12-myristate 13-acetate, that were required to induce sprouting angiogenesis (van Duinen et al., 2019). These platforms could be used, in a high throughput manner, to perfuse stromal or tumor cell conditioned medium allowing the identification of secreted factors involved in tumor angiogenesis.

Another microfluidic device using EC co-cultured with human glioblastoma cells (U87MG) or fibroblasts (control condition) in independent channel reveals that an increased number of EC sprouts invade the matrix, displaying an aberrant morphology, in response to factors secreted by tumor cells (Kim et al., 2013). Indeed, when compared to fibroblasts-induced sprouts, glioblastoma cells fail to promote formation of a perfused vascular network, only inducing immature and rarely lumenized vessels.

##### Recapitulation of the tumor vascularized microenvironment

As described above for static models, co-culture of cells in 3D hydrogels recapitulates key features of the tumor microenvironment including crosstalk between tumor and stromal cells, ECM remodeling, and storage of paracrine factors. These culture conditions have therefore been transferred to microfluidics platforms for studying: (i) direct tumor–vasculature interactions; (ii) molecular interplay between tumor–stromal cells and the vasculature (Chung et al., 2017; Miller et al., 2018; Truong et al., 2019); (iii) response to standard therapies (Sobrino et al., 2016); (iv) tumor metabolism, which is an interesting marker for cellular state and response to drugs (Sobrino et al., 2016). For this purpose, a large variety of tumor cell lines and primary cultures from many origins, including breast, renal, and colorectal cancers have been incorporated in microfluidic devices. In addition, stromal cells such as fibroblasts, MSC, or osteoblasts are also included in these models either to support capillary formation or to generate tissue-specific microenvironments.

Sobrino et al. (2016) engineered vascularized micro-tumors to assess direct tumor–vasculature interactions as well as tumor metabolism, and response to chemotactic and anti-angiogenic therapies. Their platform, based on a vasculogenesis approach, consists in two channels connected by three tissue chambers where EC, tumor cells, and fibroblasts are injected. EC formed a capillary network covered with fibroblasts that are NG2 and PDGFR-B positive, consistent with a pericyte phenotype. Various colorectal and breast tumor cell lines proliferated to form cell aggregates in close contact with the capillary network. Perfusion of this tumor-associated vascular network was demonstrated using dextran. These vascularized micro-tumors respond to chemotherapeutics, displaying reduced growth or even regression. The model established the distinction between VEGF-specific drugs that were not effective in disrupting the vascular networks and drugs against several targets (VEGFR2, PDGFR, and Tie2) that induced effective regression of the vasculature. Interestingly, tumor cells, fibroblasts, and EC that composed the micro-tumors displayed a strong metabolic heterogeneity: tumor cells were more glycolytic than the surrounding stroma, which was consistent with their known preferences for aerobic glycolysis.

One advantage of studying direct interactions within perfused vascularized micro-tumors is the possibility to easily include various stromal cells mimicking a specific tumor microenvironment. It is widely agreed that cells with stem-like properties form a fertile niche allowing tumor to grow and expand (Valkenburg et al., 2018). Indeed, in glioblastoma, a sub-population of cells with stem-like properties, glioma stem cell (GSC), has been reported as one of the major drivers of tumor recurrence, invasion of normal brain parenchyma, and resistance to therapies. Truong et al. (2019) developed a vascularized tumor-on-a-chip technology mimicking the GSC vascular niche to study EC impact on patient-derived GSC behavior. The signaling pathways governing invasion were thus identified. To this aim, EC were embedded in a hydrogel and infused within the microfluidic device to enclose the tumor region of the platform composed by patient-derived primary GSC encapsulated within Matrigel^TM^. The established microvascular network enhanced GSC migration in the hydrogel, promoted invasive morphology, and maintained GSC proliferation rates and stemness (Nestin, SOX2, CD44). Using a specific CXCR4 antagonist, the role of the CXCL12-CXCR4 signaling axis on EC-mediated GSC invasion was demonstrated. This model could be adapted to many types of cancer.

Whereas perfused vascularized micro-tumors have been engineered with various tumor cell lines (Sobrino et al., 2016; Truong et al., 2019), the use of primary cells remains challenging. Miller et al. (2018) developed a vascularized flow-directed culture system for primary human clear cell renal cell carcinoma (ccRCC) cells isolated from six patients to study the angiogenic signaling axis between ccRCC cells and EC. Collagen I containing embedded tumor spheroids were infused into the matrix channel. A retaining rod was removed from the collagen gel after polymerization to form the lumen for infusion of EC. Under a medium flow through the EC-lined lumen, capillaries were higher in all devices containing tumor cells when compared to clusters formed from normal-adjacent renal cortex. Moreover, endothelial sprouting was not limited to migration oriented toward tumor clusters but was isotropically distributed in the outlet. Indeed, computational simulation demonstrated that the biochemical gradient of angiogenic growth factors favors sprouting in the outlet region of the flow-directed microphysiological system (Miller et al., 2018). Interestingly, depending on patients, factors secreted by tumor clusters induced formation of capillaries displaying different morphologies that could reflect the inter-patient heterogeneity. This device therefore recapitulates a primary tumor cell-derived biochemical gradient.

##### Intravasation and extravasation

During the metastasis cascade, tumor cells dissociate from the primary tumor, intravasate into capillaries and survive in circulation, extravasate from the microvasculature, and form metastases in the secondary parenchyma (Chen et al., 2013, 2016). Intravasation is the transmigration of cancer cells through BM and endothelium into capillaries close to the tumor. This process is regulated by tumor cell intrinsic factors and by stromal cells present in the tumor microenvironment such as macrophages and neutrophils. To study the interplay between tumor cells and the endothelium, Wong and Searson (2014) developed microvessels formed by an EC lining method and embedded in a hydrogel containing tumor cells. Using fluorescence live cell imaging, they deciphered various tumor–endothelium interactions such as invasion and intravasation. This platform could be used to investigate the role of tumor cell–endothelium interactions and biochemical factors involved in the intravasation process. Zervantonakis et al. developed a microfluidic model to mimic cancer cell intravasation across an impaired endothelial barrier by including soluble biochemical factors or macrophages. Using an EC lining method, they demonstrated that macrophage-secreted TNF-alpha stimulated intravasation of tumor cells due to impaired endothelial barrier function (Zervantonakis et al., 2012).

Extravasation is the reverse dynamic process that involves a cascade of events including tumor–EC adhesion, initiation and formation of tumor cell protrusions into the sub-endothelial ECM, interaction with the endothelial BM, and migration through the parenchyma. The study of this process requires real-time imaging at a single cell level in a model recapitulating the human microvasculature, which is possible only with microfluidic devices. Using a microfluidic model based on vasculogenesis, Chen et al. (2016) assessed the impact of β1 integrin depletion in cancer cells on the extravasation cascade. For this purpose, individual tumor cell lines invalidated or not for β1 integrin were directly perfused into the microvessels to monitor the dynamic extravasation events. The authors deciphered multiple steps of the extravasation process and their findings highlighted a critical role for beta1 integrin in adhesion to the sub-endothelial ECM, providing new insights into the mechanisms underlying metastasis.

Microfluidic systems recapitulate the trans-endothelial migration toward a non-organ-specific ECM. However, extravasation occurs in specific tumor microenvironment composed by several cell types which could impact this process. Bersini et al. (2014) and Jeon et al. (2015) explored breast cancer cell extravasation using two microfluidic approaches in a specific bone-mimicking microenvironment (BMi). These microfluidic devices are developed by an EC lining (Bersini et al., 2014) and a vasculogenesis-based method (Jeon et al., 2015). In the device developed by Bersini et al. (2014), osteo-differentiated-human bone marrow (OD-hBM)-derived MSC were embedded in hydrogel and cultured within an independent microfluidic channel for 2–3 weeks to allow proper deposition of bone ECM. EC were thus seeded into the adjacent channel inducing formation of a continuous monolayer at the 3D hydrogel–endothelial channel interface. Finally, breast cancer cells MDA-MB 231 were injected into the hollow channel mimicking the vascular lumen and the extravasation rate was monitored. Extravasation was significantly higher in the microenvironment conditioned by osteo-differentiated cells compared to collagen hydrogel. However, in this microfluidic device, the use of an endothelial monolayer does not recapitulate the parameters of a proper capillary. Aware of the limitations of this approach, they have developed another microfluidic system based on a vasculogenesis approach. For this purpose, EC were co-embedded with hBM-MSC and OD-hBM-MSC in the central gel region of the microfluidic platform to generate a microvascular network enclosed in a BMi (Jeon et al., 2015). By perfusing individual breast tumor cell lines, the authors demonstrated that both extravasation rates and microvascular permeability were higher in the BMi compared to the muscle-mimicking microenvironment or to the unconditioned ECM. These results demonstrated a major role of the microenvironment in the extravasation process of tumor cells. This organ-specific vascularized 3D microfluidic model is an interesting tool to study cell extravasation in a stromal microenvironment and could be expanded to various cancer cells.

#### Conclusion

Microfluidic systems recapitulate many features of tumor microenvironment such as perfused and functional capillary networks, growth factor gradient, and shear stress. These models are powerful tools to study indirect and direct interactions between tumor and stromal cells with the vasculature. This approach is consistent with high resolution real-time imaging allowing to decipher each step of complex cellular processes such as intra- and extravasation (Zervantonakis et al., 2012; Chen et al., 2013, 2016). Table 2 sums up the main features and applications of 3D microfluidic models of vascularized micro-tumors. As discussed below, these systems are adaptable to high-throughput analysis and drug screening (Sobrino et al., 2016; Miller et al., 2018; Ko et al., 2019; van Duinen et al., 2019). However, these perfused vascularized micro-tumors still fail to recapitulate the whole and complex composition of *in vivo* tumors, and they should in the next future include more specifically components of the immune compartment.

**TABLE 2:** *In vitro* 3D vascularized tumors in perfused models and applications.

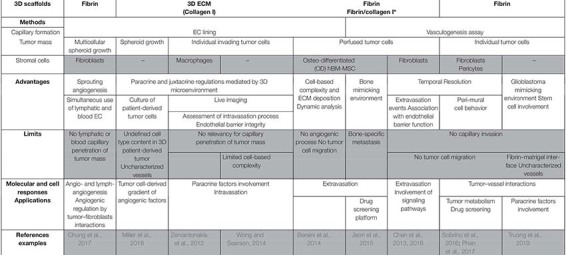

*Chen et al. (2016) is associated with fibrin/collagenI.

## Challenges and Future Applications

### High Throughput Screening of Drugs

For more than 50 years, 2D cultures of tumor cell lines have been widely used for drug screening. However, since the 2000s, many studies revealed more relevant responses to treatment of cells cultured in 3D. Tumor spheroids cultured in suspension or more recently in hydrogels have thus been proposed to bridge the gap between flat culture and *in vivo* experiments. Furthermore, vascularization as well as co-culture with stromal cells is of main interest to assess the drug delivery context. As described above, vascularized micro-tumors cultured within hydrogels (Bray et al., 2015), tissue constructs (Bourland et al., 2018; Nishiguchi et al., 2018), as well as microfluidic systems (Sobrino et al., 2016; Miller et al., 2018) are powerful platforms for cancer drug discovery and screening. However, in many cases, they are still at the proof-of-concept stage and are not designed for high throughput drug screening yet (Bray et al., 2015; Sobrino et al., 2016; Bourland et al., 2018; Miller et al., 2018; Nishiguchi et al., 2018).

Hughes’s group has engineered a perfused vascularized micro-tumor model in a 96-well plate format compatible with standard robotic and fluorescent plate readers thus creating standardized and reproducible arrays (Sobrino et al., 2016; Phan et al., 2017). Adapted for high throughput screening, each unit can be filled by various drugs. The vascularized micro-tumors can be easily extracted for further gene expression analysis. As proof-of-concept, a full-blind test of 10 FDA-approved anti-tumor compounds and two negative control compounds has been performed on the vascularized micro-tumors. Anti-angiogenic, anti-tumor drugs and placebo were successfully discriminated according to their respective effects on tumor cells or on the vasculature. More recently, aware of the need to develop standardized assays, other groups have designed microfluidic platforms suitable for high throughput anti-tumor drug screening on vascularized micro-tumors (Ko et al., 2019; Meng et al., 2019). Interestingly, Meng et al. (2019) took advantage of 3D bioprinting to accurately position cluster of tumor cells, stromal cells, and EC in their device. These new platforms share some similar characteristics essential for high throughput analyses such as a simple and quick fabrication; a miniaturized format; a single vascularized micro-tumor per well; an automated imaging.

### Toward Personalized Medicine

The development of perfused vascularized micro-tumors compatible with high throughput analysis is a major step toward personalized medicine in order to determine specific anti-tumor drugs for each individual patient.

However, up to now, personalized medicine remains challenging for many reasons: (1) the complex isolation of various cell types (tumor cells, EC, immune cells, CAF) from the same biopsy; (2) the maintenance of cell-specific functionality for a long time in culture; (3) the appropriate relative proportions of cell types; and (4) the optimal culture medium for the combined cell cultures (Sontheimer-Phelps et al., 2019). Indeed, specific isolation protocols and culture conditions are required for each cell type present in tumor biopsies. As described above, formation of *in vitro* capillaries with EC isolated from tumors is not achieved. The main improvement concerns the use of 3D-organoid technologies leading to the development of so-called “tumoroïds” that are composed by patient-derived 3D cultures of cells isolated from tumor biopsies. As previously reviewed by Drost and Clevers (2018), primary cells can be isolated from various cancer types such as colorectal, pancreatic, liver, breast, prostate, brain and bladder cancers to form these “tumoroïds.” These cells derived from living tissue are more likely to reflect the properties of native cells *in vivo.* A major step to move toward personalized medicine will be to form vascularized micro-tumors, in a high throughput manner, by co-culturing “tumoroid” and EC.

#### Induced Pluripotent Stem Cells

Recent results suggest that cancer cells remain susceptible to transcription factor-mediated reprogramming (Utikal et al., 2009). As for angiogenesis, although human induced pluripotent stem cells (iPSC) can differentiate into functional EC, they still exhibit limited expansion potential compared with human embryonic stem cells-derived EC. However, individual lines of human embryonic stem cells and iPSC are distinct and can often respond very differently to the same microenvironment cues. Thus, efficient and robust induction of EC from human pluripotent stem cells and multiple human iPSC lines or reset EC might also constitute a renewable and infinite cell source (Jahan and McCloskey, 2020; Palikuqi et al., 2020). These findings open new perspectives which should facilitate the study of epigenetic changes in human cancer and angiogenesis studies using pre-clinical complex *in vitro* 3D systems.

#### CRISPR/Cas9 Gene Editing

CRISPR-Cas9 genome editing has recently been used in tumor organoids to introduce mutations into genes commonly found mutated such as *Trp53*, *Brca1*, *Nf1*, and *Pten* in high-grade serous ovarian cancer (Lõhmussaar et al., 2020). This technology could be further used on organoids to investigate the nature and extent of intra-tumor diversification but also to study the role of mutational diversification associated with response variability to anticancer drugs between even closely related cells of the same tumor as reported by Roerink et al. (2018). In addition, although this is not the case yet, such a technology will undoubtedly be very useful to study angiogenesis in *in vitro* 3D systems.

## Conclusion

Since unperfused models constituted powerful tool for deciphering tumor–vessel interactions, perfused models are more relevant for deciphering extra- and intravasation mechanisms. However, both types of these models present limits such as (i) low representation of relevant capillaries displaying a pericyte coverage, (ii) low level of concomitant occurrence of tumor migratory processes and capillary growth for analyzing the co-option processes or the regulation of migratory strategies, and (iii) rare investigation of the interaction types between tumor mass and capillaries as well as underlying mechanisms.

A further present limit of these models is the absence of a wide panel of stromal cells since most of the studies are based on 3D microenvironment constituted by ECM and cells such as fibroblasts, MSC, macrophages, and pericytes. These is an obvious increasing need of engineering *in vitro* 3D vascularized micro-tumors for investigating the role of inflammatory cells as well as cytokines recapitulating the complex immune compartment. Moreover, another feature to consider is the EC origins and heterogeneities. As example, it has been recently reported that EC display metabolic transcriptome heterogeneity and plasticity in tumor angiogenesis (Rohlenova et al., 2020).

## Author Contributions

NB-J, CM, LM, and SG wrote the review and the tables. All authors contributed to the article and approved the submitted version.

## Conflict of Interest

The authors declare that the research was conducted in the absence of any commercial or financial relationships that could be construed as a potential conflict of interest.
